# Author Correction: Frequency and spectrum of mutations in human sperm measured using duplex sequencing correlate with trio-based *de novo* mutation analyses

**DOI:** 10.1038/s41598-024-82618-x

**Published:** 2024-12-19

**Authors:** Jonatan Axelsson, Danielle LeBlanc, Habiballah Shojaeisaadi, Matthew J. Meier, Devon M. Fitzgerald, Daniela Nachmanson, Jedidiah Carlson, Alexandra Golubeva, Jake Higgins, Thomas Smith, Fang Yin Lo, Richard Pilsner, Andrew Williams, Jesse Salk, Francesco Marchetti, Carole Yauk

**Affiliations:** 1 Department of Biology, University of Ottawa, Ottawa, ON K1N 6N5 Canada; 2 Reproductive Medicine Centre, Skåne University Hospital, Malmö, Sweden; 3 Department of Translational Medicine, Lund University, Malmö, Sweden; 4 Department of Laboratory Medicine, Lund University, Lund, Sweden; 5 Environmental Health Science and Research Bureau, Health Canada, Ottawa, Canada; 6 TwinStrand Biosciences, Inc., Seattle, WA USA; 7 Department of Obstetrics & Gynecology, Wayne State University, Detroit, MI USA

Correction to: *Scientific Reports* 10.1038/s41598-024-73587-2, published online 08 October 2024

The Competing interests section in the original version of this Article was incomplete. Furthermore, the initials ‘SG’ and ‘JJS’ have been corrected to ‘AG’ and ‘JS’, respectively, in this section.

As a result,

“JA, CY, MM, AW, FM declare no competing interests. DF, JC, SG, JH, TS, DN, FYL and JJS are employees and equity holders at TwinStrand Biosciences, Inc., or were during their contributions to this manuscript.”

now reads:

“JA, CY, DLB, HS, MM, RP, AW and FM declare no competing interests. DF, JC, AG, JH, TS, DN, FYL and JS are employees and equity holders at TwinStrand Biosciences, Inc., or were during their contributions to this manuscript.”

The original Article has been corrected.

