# Supplementary material for: Frequency and spectrum of mutations in human sperm measured using duplex sequencing correlate with trio-based de novo mutation analyses
Source: Sci Rep. 2024 Oct 8;14:23134. doi: 10.1038/s41598-024-73587-2 (PMC11461794; doi:10.1038/s41598-024-73587-2)
Supplement: Supplementary file 1 — Supplementary Material 1 [file 41598_2024_73587_MOESM1_ESM.docx]

**Supplementary Material to**

**Frequency and spectrum of somatic mutations in human sperm measured using duplex sequencing correlate with trio-based *de novo* mutation analyses**

Jonatan Axelsson, Danielle LeBlanc, Habiballah Shojaeisaadi, Matthew J Meier, Devon M. Fitzgerald, Daniela Nachmanson, Jedidiah Carlson, Alexandra Golubeva, Jake Higgins, Thomas Smith, Fang Yin Lo, Richard Pilsner, Andrew Williams, Jesse Salk, Francesco Marchetti, and Carole Yauk

**Supplementary Table S1.** Human Mutagenesis Panel targets

| **Contig** | **Start** | **End** | **Description** | **Location relative to genes** | **Gene (Genome Data Viewer NCBI)** |
| --- | --- | --- | --- | --- | --- |
| chr1 | 84597127 | 84599527 | region_208 | Genic/ intergenic | LINC01461 |
| chr2 | 40162767 | 40165167 | region_2896 | Genic | SLC8A1-AS1 |
| chr4 | 22386244 | 22388644 | region_4173 | Genic/ intergenic | ADGRA3 |
| chr6 | 155239014 | 155241414 | region_5020 | Genic | TIAM2 |
| chr7 | 11732774 | 11735174 | region_5144 | Genic | THSD7A |
| chr8 | 51513056 | 51515456 | region_5520 | Genic | PXDNL |
| chr9 | 23709463 | 23711863 | region_5739 | Genic | ELAVL2 |
| chr10 | 128969037 | 128971437 | region_784 | Intergenic | |
| chr11 | 108510787 | 108513187 | region_1111 | Genic | EXPH5 |
| chr12 | 114115043 | 114117443 | region_1355 | Intergenic | |
| chr13 | 75803912 | 75806312 | region_1501 | Genic | LMO7 |
| chr14 | 74661755 | 74664155 | region_1725 | Genic | AREL1 |
| chr15 | 46089737 | 46092137 | region_1904 | Intergenic | |
| chr16 | 51754103 | 51756503 | region_2115 | Genic/ intergenic | LOC105371257 |
| chr17 | 70672726 | 70675126 | region_2378 | Intergenic | |
| chr18 | 5749264 | 5751664 | region_2457 | Genic | MIR3976HG |
| chr19 | 31831021 | 31833421 | region_2739 | Genic/ intergenic | LINC01837 |
| chr20 | 24153684 | 24156084 | region_3388 | Genic | LINC01721 |
| chr21 | 23665976 | 23668376 | region_3515 | Intergenic | |
| chr22 | 48262370 | 48264770 | region_3703 | Intergenic | |

**Supplementary Table S2a.** Performance metrics of the assay across the different samples

| **TS ID** | **Manuscript ID** | **Raw Reads** | **Raw Reads (Million)** | **Median Insert Size (bp)** | **% Selected Bases** | **Peak Tag Family Size (SSCS)** | **Mean Duplex Depth** | **Peak Duplex Depth** | **% Mean GE to Duplex Conversion** | **Informative Duplex Bases** | **Informative Duplex Bases (Billion)** |
| --- | --- | --- | --- | --- | --- | --- | --- | --- | --- | --- | --- |
| DNA03182 | participant_1_sperm | 366 342 402 | 366 | 236 | 99,7% | 12 | 23 925 | 33 266 | 13,3% | 1 478 243 267 | 1,5 |
| DNA03183 | participant_2_sperm | 422 609 132 | 423 | 235 | 99,7% | 12 | 27 563 | 38 239 | 15,3% | 1 711 284 113 | 1,7 |
| DNA03184 | participant_3_sperm | 388 288 156 | 388 | 243 | 99,7% | 12 | 25 707 | 37 323 | 14,3% | 1 571 270 750 | 1,6 |
| DNA03185 | participant_4_sperm | 471 285 392 | 471 | 230 | 99,8% | 13 | 27 493 | 37 599 | 15,3% | 1 730 738 612 | 1,7 |
| DNA03186 | participant_5_sperm | 431 398 728 | 431 | 243 | 99,7% | 13 | 26 095 | 35 663 | 14,5% | 1 605 731 308 | 1,6 |
| DNA03187 | participant_6_sperm | 383 324 142 | 383 | 247 | 99,6% | 10 | 27 165 | 39 371 | 15,1% | 1 662 004 914 | 1,7 |
| DNA03188 | participant_1_blood | 498 369 320 | 498 | 235 | 99,8% | 14 | 28 571 | 37 022 | 15,9% | 1 787 958 086 | 1,8 |
| DNA03189 | participant_2_blood | 505 653 666 | 506 | 241 | 99,7% | 15 | 28 518 | 36 733 | 15,8% | 1 772 275 492 | 1,8 |
| DNA03190 | participant_3_blood | 399 911 138 | 400 | 233 | 99,8% | 12 | 25 376 | 33 898 | 14,1% | 1 585 922 902 | 1,6 |
| DNA03191 | participant_4_blood | 511 885 874 | 512 | 229 | 99,8% | 15 | 28 198 | 37 033 | 15,7% | 1 780 117 317 | 1,8 |
| DNA03192 | participant_5_blood | 562 685 276 | 563 | 233 | 99,8% | 17 | 28 231 | 35 898 | 15,7% | 1 777 523 729 | 1,8 |
| DNA03193 | participant_6_blood | 479 279 498 | 479 | 241 | 99,8% | 14 | 27 641 | 35 211 | 15,4% | 1 729 165 358 | 1,7 |
| DTC | TS_control_blood | 411 376 602 | 411 | 250 | 99,7% | 11 | 27 104 | 36 448 | 15,1% | 1 673 487 528 | 1,7 |

Abbreviations: DTC, DNA Technical Control; TS, TwinStrand

**Supplementary Table S2b.** Performance metrics of the assay across the different loci

| **Locus** | **min Mean Duplex Depth** | **max Mean Duplex Depth** | **mean Mean Duplex Depth** |
| --- | --- | --- | --- |
| chr1 | 24310 | 29884 | 27829 |
| chr2 | 25556 | 30750 | 28887 |
| chr4 | 21894 | 27514 | 25496 |
| chr6 | 26268 | 31120 | 29469 |
| chr7 | 18557 | 23523 | 20715 |
| chr8 | 23176 | 28596 | 26603 |
| chr9 | 20350 | 25685 | 23592 |
| chr10 | 24621 | 29195 | 27770 |
| chr11 | 25751 | 31353 | 29369 |
| chr12 | 27856 | 32512 | 30684 |
| chr13 | 24344 | 29646 | 27828 |
| chr14 | 26841 | 30747 | 29542 |
| chr15 | 21553 | 27317 | 25061 |
| chr16 | 24508 | 28503 | 27205 |
| chr17 | 21871 | 27028 | 25139 |
| chr18 | 25482 | 30037 | 28640 |
| chr19 | 24101 | 28708 | 27202 |
| chr20 | 26805 | 31538 | 29832 |
| chr21 | 16537 | 22370 | 19591 |
| chr22 | 27711 | 33141 | 30439 |
|  |  |  |  |

**Supplementary Table S3.** Locations, number, and frequency of SNV mutations across the loci in the Duplex Sequencing Human Mutagenesis panel in *blood* from the six men.

| **Location,  Chromosome** | **Type** | **SNVs** | **Total duplex bases** | **SNV MF** |
| --- | --- | --- | --- | --- |
| 1 | Genic/intergenic | 21 | 4.1 x 10^8^ | 5.1 x 10^-8^ |
| 2 | Genic | 63 | 4.2 x 10^8^ | 1.5 x 10^-7^ |
| 4 | Genic/intergenic | 53 | 3.8 x 10^8^ | 1.4 x 10^-7^ |
| 6 | Genic | 30 | 4.3 x 10^8^ | 7.0 x 10^-8^ |
| 7 | Genic | 38 | 3.1 x 10^8^ | 1.2 x 10^-7^ |
| 8 | Genic | 62 | 3.9 x 10^8^ | 1.6 x 10^-7^ |
| 9 | Genic | 56 | 3.5 x 10^8^ | 1.6 x 10^-7^ |
| 10 | Intergenic | 60 | 4.0 x 10^8^ | 1.5 x 10^-7^ |
| 11 | Genic | 26 | 4.4 x 10^8^ | 6.0 x 10^-8^ |
| 12 | Intergenic | 74 | 4.4 x 10^8^ | 1.7 x 10^-7^ |
| 13 | Genic | 21 | 4.1 x 10^8^ | 5.1 x 10^-8^ |
| 14 | Genic | 14 | 4.3 x 10^8^ | 3.3 x 10^-8^ |
| 15 | Intergenic | 49 | 3.7 x 10^8^ | 1.3 x 10^-7^ |
| 16 | Genic/intergenic | 62 | 3.8 x 10^8^ | 1.6 x 10^-7^ |
| 17 | Intergenic | 41 | 3.7 x 10^8^ | 1.1 x 10^-7^ |
| 18 | Genic | 68 | 4.2 x 10^8^ | 1.6 x 10^-7^ |
| 19 | Genic/intergenic | 57 | 4.0 x 10^8^ | 1.4 x 10^-7^ |
| 20 | Genic | 48 | 4.3 x 10^8^ | 1.1 x 10^-7^ |
| 21 | Intergenic | 41 | 3.0 x 10^8^ | 1.4 x 10^-7^ |
| 22 | Intergenic | 66 | 4.3 x 10^8^ | 1.5 x 10^-7^ |
| *TOTAL* |  | *950* | *79 x 10^8^* | *1.2 x 10^-7^* |

**Supplementary Table S4.** Locations, number, and frequency of SNV mutations across the loci in the Duplex Sequencing Human Mutagenesis panel in the *sperm* DNA of the six men.

|  |  | **SNV mutations** | | |
| --- | --- | --- | --- | --- |
| **Location,**  **Chromosome** | **Type** | **SNVs** | **Total duplex bases** | **SNV MF** |
| 1 | Genic/intergenic | 11 | 3.8 x 10^8^ | 2.9 x 10^-8^ |
| 2 | Genic | 12 | 4.0 x 10^8^ | 3.0 x 10^-8^ |
| 4 | Genic/intergenic | 6 | 3.4 x 10^8^ | 1.8 x 10^-8^ |
| 6 | Genic | 14 | 4.1 x 10^8^ | 3.4 x 10^-8^ |
| 7 | Genic | 4 | 2.8 x 10^8^ | 1.4 x 10^-8^ |
| 8 | Genic | 14 | 3.6 x 10^8^ | 3.9 x 10^-8^ |
| 9 | Genic | 9 | 3.2 x 10^8^ | 2.8 x 10^-8^ |
| 10 | Intergenic | 16 | 3.9 x 10^8^ | 4.1 x 10^-8^ |
| 11 | Genic | 3 | 4.0 x 10^8^ | 7.0 x 10^-9^ |
| 12 | Intergenic | 6 | 4.4 x 10^8^ | 1.4 x 10^-8^ |
| 13 | Genic | 10 | 3.8 x 10^8^ | 2.6 x 10^-8^ |
| 14 | Genic | 13 | 4.2 x 10^8^ | 3.1 x 10^-8^ |
| 15 | Intergenic | 3 | 3.4 x 10^8^ | 8.7 x 10^-9^ |
| 16 | Genic/intergenic | 9 | 3.7 x 10^8^ | 2.5 x 10^-8^ |
| 17 | Intergenic | 7 | 3.5 x 10^8^ | 2.0 x 10^-8^ |
| 18 | Genic | 7 | 4.0 x 10^8^ | 1.8 x 10^-8^ |
| 19 | Genic/intergenic | 10 | 3.8 x 10^8^ | 2.7 x 10^-8^ |
| 20 | Genic | 10 | 3.8 x 10^8^ | 2.7 x 10^-8^ |
| 21 | Intergenic | 5 | 2.6 x 10^8^ | 1.9 x 10^-8^ |
| 22 | Intergenic | 14 | 4.4 x 10^8^ | 3.2 x 10^-8^ |
| TOTAL |  | 184 | 7.5 x 10^9^ | 2.5 x 10^-8^ |

**Supplementary Table S5.** Subtypes of SNV base substitutions in *blood*

| **Base substitution subtype** | **Numbers** | **Mutation Frequency Mean (per bp)** | **Standard deviation  (per bp)** | **Proportion (%)** |
| --- | --- | --- | --- | --- |
| **C>A** | 131 | 3.9 x 10^-8^ | 1.2 x 10^-8^ | 14% |
| **C>G** | 59 | 1.8 x 10^-8^ | 7.7 x 10^-9^ | 6.2% |
| **C>T** | 506 | 1.5 x 10^-7^ | 2.2 x 10^-8^ | 53% |
| **T>A** | 79 | 1.7 x 10^-8^ | 7.0 x 10^-9^ | 8.3% |
| **T>C** | 118 | 2.6 x 10^-8^ | 6.5 x 10^-9^ | 12% |
| **T>G** | 57 | 1.3 x 10^-8^ | 6.5 x 10^-9^ | 6.0% |
| ***Total*** | 950 | *NA* | *NA* | *100%* |

**Supplementary Table S6.** Subtypes of base substitutions in *sperm*

| **Base substitution subtype** | **Numbers** | **Mutation frequency Mean (per bp)** | **Standard deviation** | **Proportion (%)** |
| --- | --- | --- | --- | --- |
| **C>A** | 14 | 4.3 x 10^-9^ | 2.5 x 10^-9^ | 7.6% |
| **C>G** | 16 | 5.1 x 10^-9^ | 3.4 x 10^-9^ | 8.7% |
| **C>T** | 84 | 2.6 x 10^-8^ | 5.3 x 10^-9^ | 46% |
| **T>A** | 12 | 2.8 x 10^-9^ | 2.2 x 10^-9^ | 6.5% |
| **T>C** | 47 | 1.1 x 10^-8^ | 2.5 x 10^-9^ | 26% |
| **T>G** | 11 | 2.6 x 10^-9^ | 1.3 x 10^-9^ | 6.0% |
| ***Total*** | *184* | *NA* | *NA* | *100%* |

**Supplementary Table S7.** Subtypes, numbers, and MF of indels in *blood*

| **Indels** | | | | | | | | | | | | **MNVs** | | **SVs** | |
| --- | --- | --- | --- | --- | --- | --- | --- | --- | --- | --- | --- | --- | --- | --- | --- |
| **1-2 bp** | | | | **3-20 bp** | | | | **>20 bp** | | | |  | |  | |
| **Number** | | **MF** | | **Number** | | **MF** | | **Number** | | **MF** | | **Number** | **MF** | **Number** | **MF** |
| 35 | | 4.4 x 10^-9^ | | 18 | | 2.3 x 10^-9^ | | 12 | | 1.5 x 10^-9^ | | 10 | 1.3 x 10^-9^ | 3 | 3.8 x 10^-10^ |
| **Ins** | **Del** | **Ins** | **Del** | **Ins** | **Del** | **Ins** | **Del** | **Ins** | **Del** | **Ins** | **Del** |  |  |  |  |
| 8 | 27 | 1.0 x 10^-9^ | 3.4 x 10^-9^ | 1 | 12 | 1.3 x 10^-10^ | 1.5 x 10^-9^ | 7 | 5 | 8.9 x 10^-10^ | 6.3 x 10^-10^ |  |  |  |  |

Abbreviations: Del, deletions; Ins, insertions

**Supplementary Table S8.** Subtypes, numbers, and MF of indels in *sperm*

| **Indels** | | | | | | | | | | | | **MNVs** | | **SVs** | |
| --- | --- | --- | --- | --- | --- | --- | --- | --- | --- | --- | --- | --- | --- | --- | --- |
| **1-2 bp** | | | | **3-20 bp** | | | | **>20 bp** | | | |  | |  | |
| **Number** | | **MF** | | **Number** | | **MF** | | **Number** | | **MF** | | **Number** | **MF** | **Number** | **MF** |
| 33 | | 4.4 x 10^-9^ | | 59 | | 7.9 x 10^-9^ | | 446 | | 7.0 x 10^-8^ | | 3 | 4.0 x 10^-10^ | 68 | 9.1 x 10^-9^ |
| **Ins** | **Del** | **Ins** | **Del** | **Ins** | **Del** | **Ins** | **Del** | **Ins** | **Del** | **Ins** | **Del** |  |  |  |  |
| 7 | 26 | 9.4 x 10^-10^ | 3.5 x 10^-9^ | 1 | 58 | 1.3 x 10^-10^ | 7.8 x 10^-9^ | 262 | 184 | 3.5 x 10^-8^ | 2.5 x 10^-8^ |  |  |  |  |

Abbreviations: Del, deletions; Ins, insertions


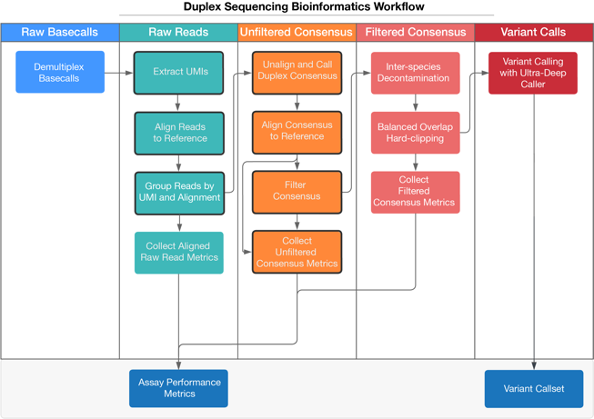


**Supplementary Figure S1.** The bioinformatics workflow used in the study


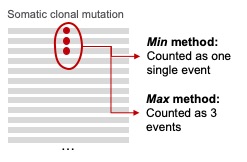


**Supplementary Figure S2.** Mutations appearing more than once in the same sample were assumed to have arisen from clonal expansion and counted only once. Each row represents a duplex consensus sequence (representing one original DNA molecule) of a specific stretch of DNA that, which is mutated or not at the specific base-pair.

**Supplementary Figure S3.** SNV mutation frequency in sperm vs in blood in the 20 different loci. R^2^ value derived from graph in Excel.

**Supplementary Figure S4.** Histograms of allele sizes for variants longer than 20 bp, with separate panels for tissues and variant types. For consistency with mutation frequency calculations, only variants with VAF < 1% are included.

**
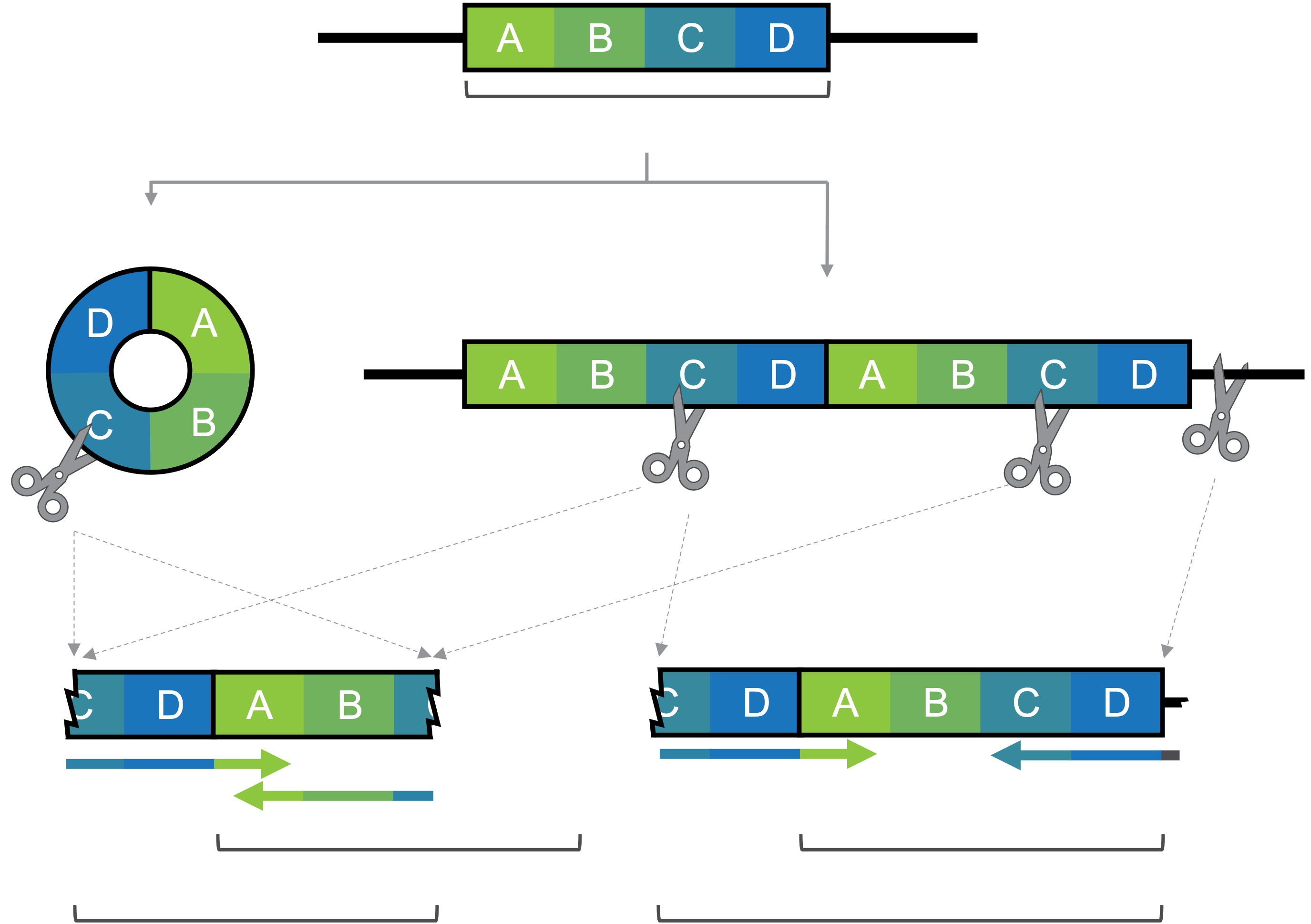
**

chromosomal tandem duplication with **D-A** junction

circular DNA with **D-A** junction

**from circle or TD**

(fragment size ≤ allele length, no duplicated sequence within fragment)

*allele length = 400 bp*

*allele length = 400 bp*

*fragment size ≤ 400 bp*

*fragment size > 400 bp*

**from TD only**

(fragment size > allele length**,** duplicated sequence within fragment)

*allele length = 400 bp*

B

C

A

**ii.**

**i.**

**ii.**

**i.**

**Supplementary Figure S5.** Schematic showing the relationship between allele length and fragment size for a D-A junction-containing molecule arising from circular DNA or a chromosomal tandem duplication*.* If a 400 bp long reference allele *ABCD* (A) forms a circular DNA (Bi), one cleavage by the fragmentation enzyme (scissors) will yield a linear fragment with length equal to the allele length of 400 bp (Ci). Two or more cleavages of the circular DNA molecule with yield fragments smaller than the allele length (not shown). A chromosomal TD of *ABCD* (Bii) can be cleaved into fragments smaller than (not shown), equal to (Ci), or larger than (Cii) the allele length of 400 bp. Note that the longer fragment (Cii) includes multiple copies of the reference allele sequences C and D.
